# Gastric-type endocervical adenocarcinoma: a case report and literature review

**DOI:** 10.3389/fonc.2024.1341068

**Published:** 2024-04-23

**Authors:** Xiao Wen, Li Yu, Xiangyu Liu, Xinjia He, Yuanyuan Zhao, Guoliang Li

**Affiliations:** ^1^ Department of Radiation Oncology, The Affiliated Hospital of Qingdao University, Qingdao, Shandong, China; ^2^ Department of Obstetrics and Gynecology, The Affiliated Hospital of Qingdao University, Qingdao, Shandong, China

**Keywords:** endocervical adenocarcinoma, gastric-type, human papillomavirus, treatment, case report

## Abstract

Gastric-type endocervical adenocarcinoma (G-EAC) represents a rare variant of cervical mucinous adenocarcinoma that is typically unrelated to human papillomavirus (HPV) infection. G-EAC exhibits highly atypical clinical presentations and characteristics, and aggressive biological behavior often leads to challenges in timely diagnosis. Here, we present a case study involving a 74-year-old Chinese woman who experienced urinary incontinence for one month. Biopsy pathology confirmed the diagnosis of G-EAC, revealing stage IVa by imaging examinations. The patient subsequently underwent three cycles of chemotherapy, followed by adjuvant radiotherapy and surgical excision of residual tumor foci. This comprehensive treatment approach yielded a favorable survival outcome. For patients with advanced G-EAC, a multimodal therapeutic approach holds promise and warrants further exploration.

## Introduction

Cervical cancer is the most prevalent malignancy affecting the female reproductive system. While the majority of cases are associated with persistent HPV infection, a subset of cervical cancers, predominantly adenocarcinomas, occur independently of HPV infection. Approximately 20-25% of cervical cancers are classified as endocervical adenocarcinomas (EAC), and the incidence of cervical adenocarcinoma has been increasing in recent years (1). The 2020 World Health Organization (WHO) classification categorizes EAC into HPV-associated and HPV-independent adenocarcinoma to emphasize the morphological and pathogenetic correlation. G-EAC is the most common type of HPV-independent adenocarcinoma, second only to usual-type endocervical adenocarcinoma (UEA) in terms of incidence, and the second most common primary adenocarcinoma of the cervix (2). G-EAC exhibits a relatively higher prevalence in Japan, while being considered rare in Western countries. Notably, Kojima et al. (3) reported a lower 5-year disease-specific survival rate of 30% for GAS compared to 77% for usual-type UEA. This uncommon neoplasm demands attention due to its aggressive behavior and absence of HPV association. And this may result in a probable delay in diagnosis and a worse prognosis (4, 5). In this report, we present the case of a patient with G-EAC who underwent three cycles of chemotherapy followed by radiotherapy to the cervix and regional lymph nodes. Subsequently, the patient underwent excision of the residual cervical lesion, resulting in a substantial improvement in long-term survival. This report underscores the important clinical impact of this multimodal treatment approach for G-EAC patients.

## Case presentation

A 74-year-old postmenopausal woman presented to the hospital with a complaint of urinary incontinence persisting for more than one month. The patient also reported symptoms of vulvar pruritus without associated skin excoriation. There was no accompanying abdominal pain or distention, and the vaginal examination revealed no abnormal discharge, bleeding, or fluid. The patient was previously in poor health, was diagnosed with thrombocytopenic purpura, and underwent coronary stenting for myocardial infarction.

**Figure 1:**
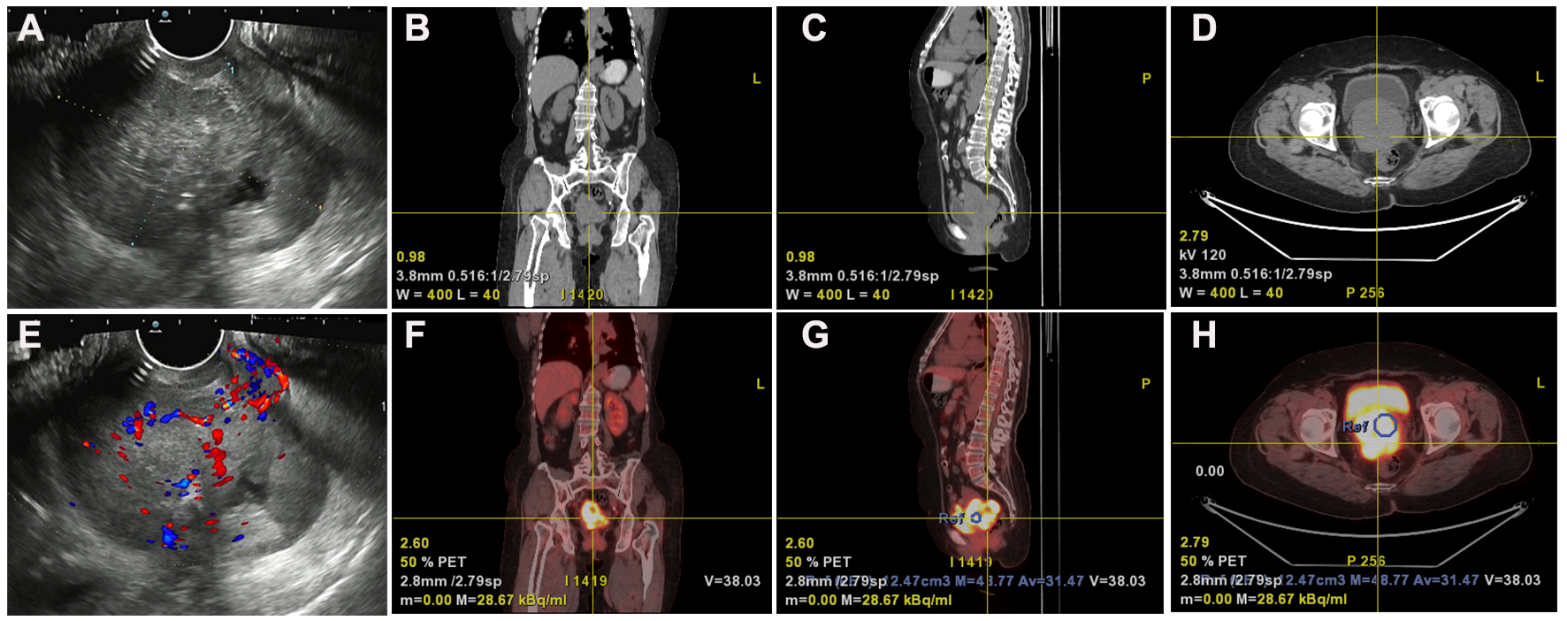
Ultrasound and PET-CT imaging revealed anatomical localization and metabolic activity. **(A, E)** Gynecologic ultrasound reveals an irregularly shaped, Multinodular cervical mass lesion measuring approximately 7.9 cm×6.8 cm×5.7 cm with partially indistinct boundaries, suggestive of confluence of multiple nodules. **(B–D, F–H)** PET-CT imaging demonstrates cervical soft tissue mass with necrosis, involvement of the uterine corpus and vagina, indistinct boundaries with bladder and rectum, and increased metabolic activity (SUVmax 16.8) in the Case of G-EAC.

**Figure 2:**
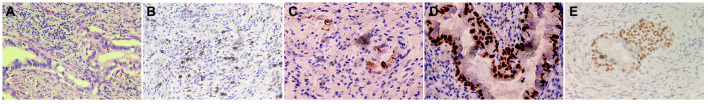
Histopathological findings in the patient of G-EAC. **(A)** H&E staining demonstrates the histological features of G-EAC (400X). The tumor cells exhibit irregular glandular structures, invasive growth pattern, and cytoplasmic features characterized by transparency and a foamy appearance. The nuclei display irregular distribution along the basal portion of the glands. **(B–D)** IHC staining of the primary tumor cells showed that Ki-67, MUC6 and PAX8 were positive expression (400X). **(E)** P53 mutations were observed in the case.

Transvaginal ultrasonography revealed a cervical mass with dimensions approximately 7.9 cm×6.8 cm× 5.7 cm in October 2021. The lesion demonstrated an irregular, lobulated shape with indistinct boundaries in certain areas, suggesting the presence of multiple nodules merging together (**Figures 1A, E**). PET-CT revealed a soft tissue density mass with necrosis in the uterine cervix invading the uterine body upward and invading the vagina downward, with indistinct demarcation between the bladder and rectum at some level and increased metabolism and a SUVmax of 16.8; also revealed were multiple enlarged lymph nodes in the retroperitoneal inferior vena cava parietal and bilateral iliac vessel travel areas; cervical cancer with metastasis to the above lymph nodes and invasion of the uterine body, vagina, adjacent bladder, and rectum was apparent (**Figures 1B–D, F–H**). The patient’s histological classification was subsequently confirmed as G-EAC through biopsy pathology. H&E staining revealed the presence of irregular glandular structures in the tumor cells, accompanied by an invasive growth pattern. The nuclei display irregular distribution along the basal portion of the glands. The cytoplasm exhibited distinctive features, characterized by a transparent and foamy appearance (**Figure 2A**). Simultaneously, immunohistochemistry (IHC) was used to analyze the expression of Ki-67 (1:150 dilutions; #ZM-0167, Zhong Shan Golden Bridge Biological Technology Inc.), Mucin 6 (MUC6) (1:200 dilutions; #ZM-0396, Zhong Shan Golden Bridge Biological Technology Inc.), Paired Box 8 (PAX8) (1:150 dilutions; #ZM-0468, Zhong Shan Golden Bridge Biological Technology Inc.) and p53 (1:150 dilutions; #ZM-0408, Zhong Shan Golden Bridge Biological Technology Inc.).

**Figure 3:**
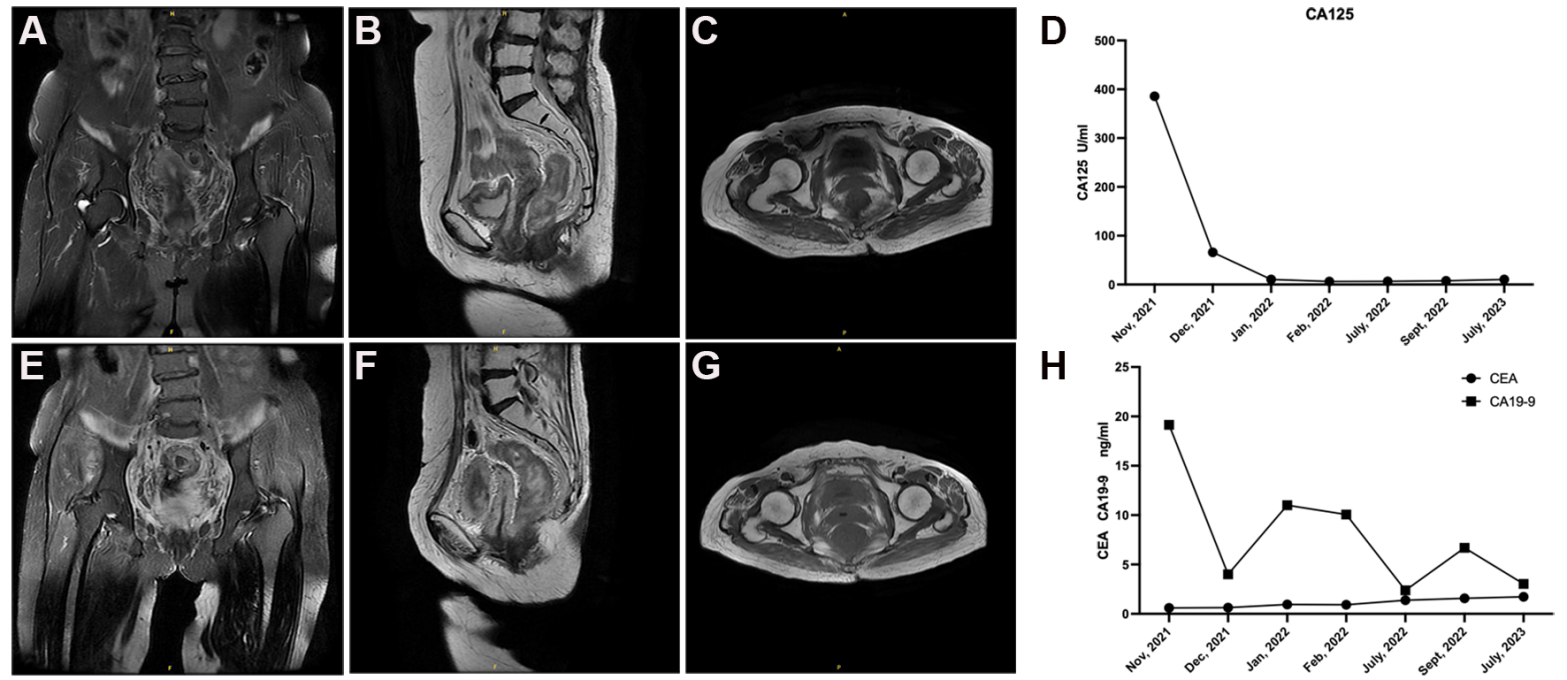
Follow-up pelvic MRI and tumor marker results in the patient with G-EAC. **(A–C)** The patient exhibited a substantial reduction in lesion size on MRI following radiotherapy and chemotherapy. The maximum cross-sectional dimension was measured to be approximately 1.3 cm×2.0 cm. **(E–G)** Regular pelvic MRI follow-up examinations revealed no signs of tumor recurrence in the postoperative period for this patient. **(D)** Prominent reduction in CA125 levels observed in the patient's blood after commencement of chemotherapy, demonstrating sustained lower levels throughout subsequent follow-up period. **(H)** CA19-9 and CEA levels fluctuated within the normal range, accompanied by a remarkable decrease in CA19-9 subsequent to radiotherapy and chemotherapy.

The results showed that Ki-67, MUC6, and PAX8 were positive, while p53 was evaluated as a “wild type” in the case (**Figures 2B–E**). Additionally, given the absence of lip and finger pigmentation, gastrointestinal polyps, significant positive features in postoperative pathology, or any pertinent family history, Peutz-Jeghers syndrome (PJS) can be reasonably excluded. We staged the patient according to the clinical staging criteria of the International Federation of Gynecology and Obstetrics (FIGO) as stage IVa (6). Considering the advanced stage of the tumor and the low platelet (PLT), the patient was not suitable for surgery at this time. The patient subsequently received 3 cycles of chemotherapy with albumin-paclitaxel combined with cisplatin from November 2021 to January 2022. Intensity-modulated radiotherapy (IMRT) was administered to both the cervix and regional lymph nodes, comprising an external beam radiotherapy dose of 52 Gy delivered in 25 fractions. Additionally, intracavity brachytherapy was performed, providing a dose of 23 Gy over 4 fractions. The total dose at point A was 84.2 Gy. Upon outpatient review, the patient demonstrated a remarkable decrease in lesion size as observed on Magnetic Resonance Imaging (MRI) after undergoing radiotherapy and chemotherapy. The maximum cross-sectional dimension was measured to be approximately 1.3 cm × 2.0 cm (**Figures 3A–C**). Regular pelvic MRI follow-up examinations revealed no signs of tumor recurrence in the postoperative period for this patient (**Figures 3E–G**). Significant reduction in CA125 levels was observed in the patient’s blood following the initiation of chemotherapy, showcasing sustained lower levels throughout the subsequent follow-up period (**Figure 3D**). Furthermore, carbohydrate antigen 19-9 (CA19-9) and carcinoembryonic antigen (CEA) levels exhibited fluctuations within the normal range, accompanied by a noteworthy decrease in CA19-9 subsequent to the administration of radiotherapy and chemotherapy (**Figure 3H**). This significant response highlights the efficacy of the combined treatment approach in managing the tumor burden in this individual. In accordance with the RECIST 1.1 (7) efficacy evaluation criteria, treatment response was classified as a partial response (PR). Subsequently, the patient underwent an open total hysterectomy and bilateral adnexal resection in September 2022. Intraoperatively, several observations were made: the uterine body appeared normal in size with a smooth surface, bilateral sacral ligaments were visibly shortened and displayed poor elasticity, the rectouterine pouch had disappeared, and no apparent abnormalities were detected in the bilateral ovaries or fallopian tubes. Postoperatively, the patient underwent regular follow-up examinations every 4 months and was continuously monitored until July 2023. No evidence of recurrence or metastasis was detected during the follow-up period.

## Discussion

Cervical cancer is one of the most common malignancies that can arise from the female genital tract and is the third most common malignancy affecting women worldwide (8). Cervical cancer can be broadly classified as adenocarcinoma or squamous cell carcinoma (SCC). As the level of screening for SCC in the general population has increased, the incidence of SCC has gradually decreased, whereas the incidence of adenocarcinoma has increased each year. Most adenocarcinomas are associated with HPV infection, called HPVA, and include usual-type, villoglandular, mucinous and invasive stratified mucin-producing carcinoma (iSMILE) subtypes. There is also a subset of adenocarcinomas not associated with HPV infection, called NHPVA, which includes endometrioid adenocarcinoma, G-EAC, clear cell adenocarcinoma, serous and mesonephric carcinoma, and invasive adenocarcinoma not otherwise specified (NOS). G-EAC accounts for approximately 10-15% of cervical adenocarcinomas worldwide (9) and is a mucinous adenocarcinoma with gastric-type differentiation and morphological features similar to those of the pyloric gland epithelium. The G-EAC is not related to high-risk HPV infection and lacks specific clinical symptoms; concealed lesions make sampling difficult; screening and biopsy positivity rates are low; and the pathological morphological characteristics of G-EAC are similar to those of benign lesions, but the biological characteristics are highly malignant, bringing great challenges to the diagnosis of G-EAC. The preoperative diagnosis rate is low, and patients are easily misdiagnosed and often never diagnosed, thus delaying treatment and seriously affecting patient prognosis. To ensure the exclusion of cervical adenocarcinoma in patients presenting with pelvic masses, ascites, and cervical lesions, it is recommended to perform a deep cervical biopsy. This procedure plays a crucial role in confirming or ruling out the presence of cervical adenocarcinoma (10). Precursor lesions are essential for cancer prevention and early detection, and the precursors of G-EAC include lobular endocervical glandular hyperplasia (LEGH), atypical LEGH, and gastric-type adenocarcinoma *in situ* of the uterine cervix, the latter two of which share common genetic features, such as 1p deletion and 3q acquisition (11). Alterations in TP53, STK11, CDKN2A, ATM, and NTRK3 have been found to be significantly more common in G-EAC (12). In addition, G-EAC is closely linked to PJS, characterized by benign hamartomatous polyps in the gastrointestinal tract, hyperpigmented macules in the oral mucosa, and a 10- to 18-fold escalation in the likelihood of developing tumors in the gastrointestinal tract and breast (13). Approximately 10% of patients with G-EAC have PJS, which may be associated with mutations in the STK11 gene (14, 15). These findings suggest that targeted sequencing assays hold potential as valuable diagnostic and prognostic tools in the management of G-EAC.

Contact vaginal bleeding is a typical symptom of cervical cancer, but the manifestations of G-EAC symptoms are relatively diverse; contact vaginal bleeding is rare, mucus-like or watery vaginal discharge is relatively common, and abdominal discomfort and lower abdominal pain are the first symptoms. In our patient, urinary incontinence was the main symptom. Therefore, to be more alert for the diagnosis, attention must be given to symptoms other than vaginal bleeding. On clinical examination, patients with G-EAC may present with an enlarged cervix, known as a “barrel” cervix; however, on colposcopy, some patients with G-EAC may not show abnormalities in appearance. Both CT and transvaginal ultrasound in our patient showed an enlarged and poorly contoured cervix. Considering the absence of specific symptoms and clinical examination findings in G-EAC, it is difficult to diagnose this disease with ultrasound, computed tomography, or other imaging techniques. Pathological assessment is the ultimate means of confirming the diagnosis of G-EAC. In general, G-EAC is difficult to recognize, with a leakage rate of up to 34%. Routine cytology, even colposcopy-guided puncture biopsy, endocervical curettage, cervical scraping, or other histologic tests, can make it difficult to detect the presence of lesions. Even when cervical conization tissue is used when necessary, deeper tissue is often needed to provide a good diagnosis.

Li et al. (16) found that G-EAC was detected in cervical biopsies in 50.7% of 185 biopsies, whereas it was detected in 100% of 14 cervical conization specimens. In an analysis of 26 G-EAC patients, Gilks et al. (17) found that 12 patients (46.2%) were diagnosed on the basis of cervical biopsy or endocervical smear specimens, whereas the remaining 14 patients were not diagnosed until pathology after resection. Similarly, several other studies found that G-EAC was successfully diagnosed preoperatively in 44.4% (8/18) and 41.2% (7/17) of patients, with the remainder being diagnosed after hysterectomy (18, 19). Nakamura et al. studied 322 patients with cervical cancer and found that it was difficult to identify G-EAC via preoperative biopsy, but several specific clinical manifestations (e.g., water discharge and lower abdominal discomfort), high serum CA 19-9 levels, and immunohistochemistry with HIK1083 and MUC6 staining could help in the diagnosis of cervical cancer (20). In our patient, the pathological diagnosis was only poorly differentiated adenocarcinoma according to the initial cervical biopsy, and G-EAC was confirmed by postoperative pathology. Considering the high risk of underdiagnosis of G-EAC, immunohistochemistry can be used as an adjunctive tool to assist in its diagnosis. On immunohistochemical staining, G-EAC usually stains for Estrogen Receptor (ER) and Progesterone Receptor (PR), and since G-EAC is not associated with HPV infection, the alternative HPV marker p16 is usually negative in G-EAC patients. Cytokeratin (CK7)、hepatocyte nuclear Factor 1 beta (HNF1β), carbonic anhydrase IX (CA IX)、 caudal type homeobox 2 (CDX2)、CK20、PAX8 were mostly positive focally or diffusely, with 68-80% of these results being positivity for PAX8, which is of value in identifying tumors that originate from the pancreatic bile duct (21). Approximately 50% of patients exhibit p53 mutant expression and a low Ki67 proliferation index, usually < 40%. MUC6 and HIK1083 are classical immune markers of gastric-type mucus, MUC6 has high sensitivity but low specificity, and HIK1083 has high specificity; moreover, the combination of the two can improve the accuracy of the test (3, 22). Our patient presented with p16 (-), ER (90%), vimentin (partial +), Pax-8 (+), CK7 (+), CK20 (-), K1-67 (+, 10%), CDX-2 (-), MUC6 (+), MUC5AC (+), SATB2 (-) and a wild type p53, showing general consistency with the literature and possibly facilitating further clarification of the diagnosis of G-EAC. G-EAC patients can have elevated tumor marker levels; approximately 1/2 or more patients with G-EAC have elevated serum CA 19-9, approximately 1/3 have elevated serum CA-125, and elevated CA-125 mostly indicates the presence of abdominopelvic metastases (20). In addition, it has also been shown that CEA levels are elevated in G-EAC patients and can be well differentiated from those in clear cell carcinoma patients (23). Our patient had elevated CA-125 levels, while her CEA and CA19-9 levels were within the normal range.

Since G-EAC is relatively rare, most of the clinical cases are reported as individual cases or small cases, and there is a lack of prospective clinical studies. There is no standard treatment thus far, and there are no treatment recommendations specific for G-EAC in various guidelines; rather, the guidelines refer to UEA and SCC. However, G-EAC exhibits distinct clinical and pathological characteristics and a markedly different prognosis than UEA and SCC. G-EAC displays enhanced invasiveness, and patients are often diagnosed at advanced stages, suggesting that they are prone to lymphatic and neural involvement and distant metastasis. Our patient was classified as stage IVa due to tumor invasion of the bladder and rectum and underwent radiotherapy followed by hysterectomy and bilateral adnexal resection due to residual tumor. Compared with UEA, G-EAC has a greater recurrence rate (40% *vs*. 14.6%) (24), a lower five-year survival rate (32% *vs*. 70%) (3); moreover, G-EAC is prone to distant metastases and has an overall worse prognosis. The prognosis was related to the stage of the tumor, the presence or absence of parametrial invasion, the condition of the incision margin, the presence or absence of metastasis and the treatment method. A study showed that programmed death ligand 1 (PD-L1)-positive G-EAC patients had a worse prognosis than PD-L1-negative patients in terms of disease progression-free survival and overall survival (25).

In conclusion, our case presentation provides a comprehensive understanding of the diagnosis and treatment approaches for G-EAC. This work highlights the unique clinical and pathological features of G-EAC, emphasizing its distinct characteristics compared to other types of cervical cancer. By increasing awareness and improving diagnosis, we can enhance patient outcomes. The combination of paclitaxel and platinum-based chemotherapy appears to be a suitable regimen for advanced G-EAC patients, as demonstrated in this case. However, it is essential to consider the potential development of drug resistance. Adjuvant radiotherapy can further reduce the risk of local recurrence, and subsequent surgery may be considered to resect residual tumor foci after comprehensive chemo- and radiotherapy approaches have been implemented. While our findings provide valuable insights, it is important to acknowledge the limitations of this study, including the small sample size and lack of a control group. Therefore, further research with larger cohorts and longer follow-up periods is necessary to validate the effectiveness of the proposed treatment approach and establish optimal therapeutic standards for G-EAC. Currently, there are no established therapeutic standards for G-EAC, and it is crucial to explore safe and effective comprehensive treatment approaches while adhering to the guidelines for cervical cancer management. With continued research and clinical studies, we can advance our understanding and improve outcomes for patients with G-EAC.

## Data availability statement

The original contributions presented in the study are included in the article/supplementary material. Further inquiries can be directed to the corresponding authors.

## Ethics statement

The studies involving humans were approved by Affiliated Hospital of Qingdao University. The studies were conducted in accordance with the local legislation and institutional requirements. The participants provided their written informed consent to participate in this study. Written informed consent was obtained from the individual(s) for the publication of any potentially identifiable images or data included in this article.

## Author contributions

XW: Writing – original draft, Writing – review & editing, Data curation. GL: Writing – review & editing, Visualization. YZ: Writing – review & editing. XL: Writing – review & editing, Investigation. LY: Writing – review & editing, Data curation. XH: Writing – review & editing, Visualization.
